# LAG-3 Expression Predicts Outcome in Stage II Colon Cancer

**DOI:** 10.3390/jpm11080749

**Published:** 2021-07-30

**Authors:** Gaëlle Rhyner Agocs, Naziheh Assarzadegan, Richard Kirsch, Heather Dawson, José A. Galván, Alessandro Lugli, Inti Zlobec, Martin D. Berger

**Affiliations:** 1 Department of Medical Oncology, Inselspital, Bern University Hospital, University of Bern, 3010 Bern, Switzerland; Gaelle.Rhyner@h-fr.ch; 2 Department of Medical Oncology, HFR Fribourg Hospital, 1708 Fribourg, Switzerland; 3 Division of Pathology and Lab Medicine, University of Toronto, Toronto, ON M5G 1X5, Canada; nassarz1@jhmi.edu (N.A.); Richard.Kirsch@sinaihealth.ca (R.K.); 4 Department of Pathology, The Johns Hopkins University School of Medicine, Baltimore, MD 21212, USA; 5 Institute of Pathology, University of Bern, 3008 Bern, Switzerland; heather.dawson@pathology.unibe.ch (H.D.); jose.galvan@pathology.unibe.ch (J.A.G.); alessandro.lugli@pathology.unibe.ch (A.L.); inti.zlobec@pathology.unibe.ch (I.Z.)

**Keywords:** biomarker, LAG-3, immune checkpoint, colon cancer, survival

## Abstract

Introduction: LAG-3 is an inhibitory immune checkpoint molecule that suppresses T cell activation and inflammatory cytokine secretion. T cell density in the tumor microenvironment of colon cancer plays an important role in the host’s immunosurveillance. We therefore hypothesized that LAG-3 expression on tumor-infiltrating lymphocytes (TILs) predicts outcome in patients with stage II colon cancer. Patients and Methods: Immunohistochemical staining for LAG-3 was performed on tissue microarrays (TMAs) of formalin-fixed paraffin-embedded tissue from 142 stage II colon cancer patients. LAG-3 expression was assessed in TILs within both the tumor front and tumor center and scored as either positive or negative. The primary endpoint was disease-free survival (DFS). Results: In patients diagnosed with stage II colon cancer, the presence of LAG-3 expression on TILs was significantly associated with better 5-year DFS (HR 0.34, 95% CI 0.14–0.80, *p* = 0.009). The effect on DFS was mainly due to LAG-3-positive TILs in the tumor front (HR 0.33, 95% CI 0.13–0.82, *p* = 0.012). Conclusion: Assessment of LAG-3 might help to predict outcomes in patients with stage II colon cancer and potentially identify those patients who might benefit from adjuvant chemotherapy. Therefore, LAG-3 may serve as a prognostic biomarker in stage II colon cancer.

## 1. Introduction

Colon cancer is a major cause of cancer-associated death worldwide and its global incidence is continuously increasing [1,2]. The prognosis of patients with colon cancer is mainly dependent on the stage according to the TNM classification system [3]. While patients diagnosed with stage I colon cancer have an excellent outcome, those with stage IV disease have a low chance of cure and a significantly worse survival [4,5]. Interestingly, some patients with stage II colon cancer, especially the ones with a pT4b tumor (stage IIC), have a worse outcome compared with those with a pT1 or pT2 N+ tumor (stage IIIA and IIIB). In fact, one might assume that a node-positive disease indicates a more aggressive tumor biology translating into a poorer clinical outcome [4]. According to current guidelines, adjuvant chemotherapy is indicated for stage III colon cancer and for stage II disease with additional clinicopathological risk factors [6]. Despite curative surgery and adjuvant chemotherapy, relapses occur and pose significant challenges for our health care system. However, some patients will not benefit from adjuvant chemotherapy because they have already been cured by surgery alone [7]. Therefore, new prognostic and predictive biomarkers have to be developed to define subgroups of patients, especially those with low- or standard risk stage II cancer who have a high chance of relapse and may derive the most benefit from chemotherapy [8].

Recently, substantial progress has been made in understanding the role of immune cell infiltration in colon cancer, which has led to the development of the Immunoscore in stage I–III colon cancer, based on the quantification of CD3+ and CD8+ lymphocytes in the tumor and its invasive margin [9]. Remarkably, this scoring system predicts disease-free and overall survival even more precisely than the TNM classification and might therefore guide our treatment decisions in the future [9]. Moreover, the identification of immune-related biomarkers on tumor-infiltrating lymphocytes (TILs) might help to predict prognosis and direct clinical decisions. Recent trials demonstrated that the presence of immune-related biomarkers such as PD-1/PD-L1 on tumor-infiltrating cells might serve as prognostic biomarkers in colorectal cancer [8,10]. The lymphocyte-activation gene 3 (LAG-3 or CD223) is another inhibitory immune-related molecule that is expressed on T cells, especially on activated CD8+ and CD4+ T cells, but also on B cells and dendritic cells, which may act in synergy with the PD-1/PD-L1 pathway [11,12]. LAG-3 mainly binds to the major histocompatibility complex II (MHC II), and thus prevents the interaction of the MHC II with the T cell receptor (TCR) on CD4+ T cells, resulting in decreased CD4+ activity. Another ligand of LAG-3 is Galectin-3, which is mainly expressed on epithelial and immune cells and preferentially binds to LAG-3 on CD8 cells [13]. The protein liver sinusoidal endothelial cell lectin (LSECtin) is a further potential ligand that binds to LAG-3 [14,15]. Upregulation of LAG-3 on immune cells downregulates T cell expansion and cytokine secretion, and thus contributes to an immunosuppressive microenvironment [16,17].

Given the growing interest in the role of LAG-3 in cancer, we sought to evaluate the presence of LAG-3 on tumor-infiltrating lymphocytes (TILs) in the tumor center and tumor front and to assess its impact on outcomes in stage II colon cancer.

## 2. Patients and Methods

### 2.1. Patient Cohort

Between 1992 and 2010, patients at the Mount Sinai University Hospital in Toronto, Canada with curatively resected stage II colon cancer in which archival material was available were consecutively included in this retrospective study. Patients with rectal cancers were excluded from our analysis.

A histopathological review was performed according to the 6th edition of the TNM classification system. Clinical data were obtained from patient records. The baseline characteristics comprised age at diagnosis, gender, tumor location, pT stage, tumor grade and lymphatic and venous vessel invasion. In addition, tumor budding, considered as a supplementary prognostic factor, was scored according to the International Tumor Budding Consensus Conference 2016 [18]. Moreover, the mismatch repair (MMR) status was determined by immunohistochemistry. The study was approved by the research ethics board of the Mount Sinai Hospital (nr 13-0136).

### 2.2. Next-Generation Tissue Microarray (ngTMA^®^) Construction

H&E-stained (hematoxylin and eosin-stained) whole slides of each case were digitized using a slide scanner (3DHistech, P250, Hungary). Each scan was annotated twice using a 0.6 mm tool in four different regions of interest: tumor center (encompassing mostly tumor epithelium), tumor front (targeting 50% tumor and 50% stromal areas at the invasion front), and tumor stroma (including largely stromal areas at the invasion front with only little, if any tumor). This produced 11 ngTMA^®^ blocks with six cores per tumor.

### 2.3. Immunohistochemistry

2.5 μm ngTMA^®^ sections were mounted onto glass slides, dried and baked at 60 °C for 30 min prior to use. All immunostainings were performed by automated staining using Bond RX (Leica Biosystems, Muttenz, Switzerland). All slides were dewaxed in Bond dewax solution (product code AR9222, Leica Biosystems). Heat-induced epitope retrieval at pH 9 in Tris buffer base (code AR9640, Leica Biosystems) followed for 30 min at 95 °C. LAG-3 rabbit monoclonal antibody (Cell Signaling, clone D2G4O Ref 15372) was diluted 1:200 and incubated for 30 min at room temperature. Then, the samples underwent incubation with HRP (Horseradish Peroxidase)-polymer for 15 min and were subsequently visualized using 3,3-Diaminobenzidine (DAB) as brown chromogen (Bond polymer refine detection, Leica Biosystems, Ref DS9800) for 10 min. Finally, the samples were counterstained with hematoxylin, dehydrated and mounted with Tissue-Tek^®^ Glas™ Mounting Media (Sakura). Slides were scanned and photographed using Pannoramic 250 (3DHistech). The immunostainings for the mismatch repair proteins were performed using the VENTANA MMR IHC Panel and the VENTANA BenchMark automated staining system (Roche Diagnostics, Mannheim, Germany) according to the manufacturer’s instructions. The following antibodies were used: anti-MLH1 (mouse, clone M1, Roche Diagnostics, Ref 8504946001), anti-MSH2 (mouse, clone G219, Roche Diagnostics, Ref 8504946001), anti-MSH6 (rabbit, clone SP93, Roche Diagnostics, Ref 8504946001) and anti-PMS2 (mouse A16-4, Roche Diagnostics, Ref 8504946001).

### 2.4. Evaluation of Immunohistochemistry

**Figure 1:**
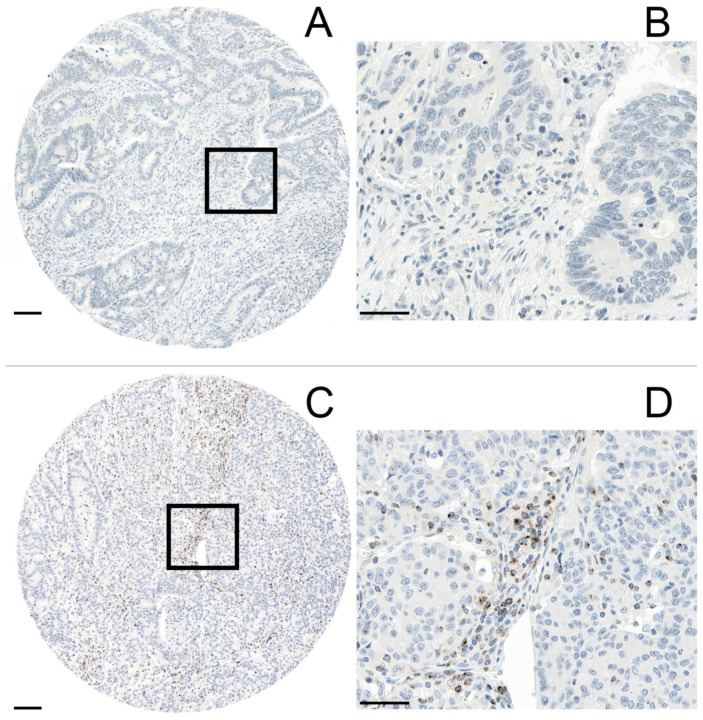
(**A**,**B**): Absence of immunohistochemical staining for LAG-3 on TILs (magnification: A 5× and B 40×). (**C**,**D**): TILs expressing LAG-3 (magnification C 5× and D 40×). Scale bar (**A**,**C**): 100 µm. Scale bar (**B**,**D**): 50 µm. All images (**A**–**D**) represent the tumor center.

All TMA cores for each individual case were evaluated for the presence or absence of LAG-3 immunohistochemical staining (G.R). Consistent with Sobottka et al., LAG-3 expression on TILs within both the tumor front and tumor center was dichotomously scored as either positive or negative [19]. Representative images are outlined in Figure 1A–D. LAG-3 positivity on TILs was defined as membranous staining of any intensity regardless of the number of LAG-3 positive immune cells (≥1), whereas the absence of any staining was determined as LAG-3 negative. We reported the scores for LAG-3 positive TILs as absolute numbers and used the maximum score of all analyzed tissue cores from each patient. The tumor front was defined as the area where the most advancing cancer cells reached the edge of the tumor. In a control set of normal, non-neoplastic colon tissues, no LAG-3 positivity could be observed. Mismatch repair deficiency (dMMR) was defined as the loss of nuclear expression of at least one of the four MMR proteins (MLH1, MSH2, MSH6 and PMS2) in the tumor cells in the presence of an internal positive control such as lymphocytes, normal epithelium or fibroblasts in the close vicinity of the tumor. Focal weak and dotted nuclear staining were considered negative. Retained nuclear expression of all MMR proteins was determined as mismatch repair proficiency (pMMR) [20].

### 2.5. Statistical Analysis

Correlations between LAG-3 expression in TILs within the tumor center/front and categorical variables were tested using the chi-square test. Continuous or ordinal variables were analyzed with the Kruskal-Wallis or Wilcoxon rank sum test. Disease-free survival (DFS) analysis was performed with Kaplan-Meier survival curves and log-rank test. Hazard ratios and 95% confidence intervals (CIs) were used to determine the effect of each variable on outcome, using Cox regression analysis. DFS was calculated from the time of surgery to local or distant recurrence or death. A *p*-value < 0.05 was considered statistically significant. Statistical analysis was carried out by SPSS version 26 (United States).

## 3. Results

### 3.1. Patients Characteristics

Our study population comprised 142 patients with curatively resected stage II colon cancer. The median age of the patients was 70 years (range, 24–98 years). 42.2% (*n* = 60) of the patients were female and 57.8% (*n* = 82) male. 85.8% (*n* = 121) of the study cohort had a pT3 tumor, whereas 14.2% (*n* = 20) of the patients presented with a pT4 tumor. 91% of the tumors (*n* = 122) were well (G1) or moderately (G2) differentiated and 9% (*n* = 12) presented with a G3 grading. 87.4% (*n* = 118) of the tumor specimens displayed no extramural venous invasion (V0), while vascular invasion could be observed in 12.6% (*n* = 17). There was a slight predominance of left- as compared to right-sided tumors (52.5%, *n* = 73 versus 47.5%, *n* = 66). In total, 124 patients (87.3%) had more than 12 lymph nodes examined, whereas the lymph node yield was less than 12 in 12.7% of the patients (*n* = 18). From 134 evaluable tumor tissue samples, 75.4% (*n* = 101) were MMR-proficient, whereas 24.6% (*n* = 33) were MMR-deficient (Supplementary Table S1). The median tumor budding count was 10 (0–74 buds). The 5-year DFS rate of the cohort was 85%.

### 3.2. LAG-3 Expression on TILs and Its Correlation with Clinicopathological Characteristics

69% (*n* = 98) of all patients exhibited LAG-3 expression on tumor-infiltrating lymphocytes.

No significant correlation could be observed between LAG-3 expression on TILs and age, gender, pT stage, grade, vascular invasion, tumor location or tumor budding. Interestingly, the percentage of MMR-deficient colon cancers was higher if LAG-3 positive TILs were present in the tumor front or center. Conversely, a lower ratio of MMR-deficient colon cancers was observed in the absence of LAG-3 positive TILs (*p* = 0.034). Additionally, LAG-3 expression on TILs in the tumor center was associated with better differentiation (grade 1, *p* = 0.021) (Table 1 and Table 2).

### 3.3. LAG-3 Expression on TILs and Its Association with DFS

**Figure 2:**
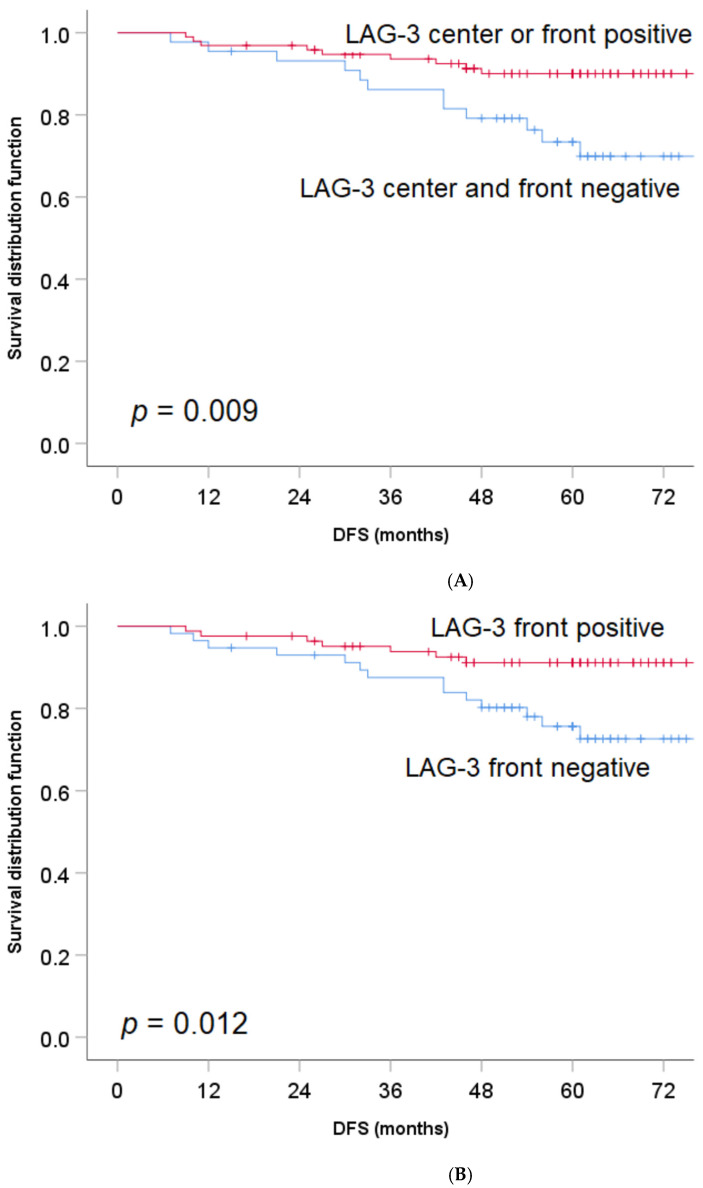

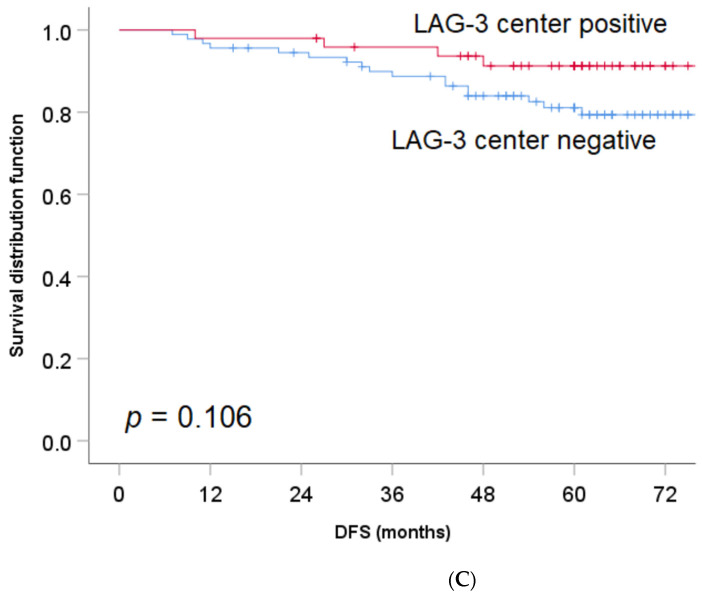
(**A**): The impact of LAG-3 expression on TILs on DFS in patients with stage II colon cancer (tumor center and tumor front). (**B**): LAG-3 expression on TILs at tumor front and its effect on DFS in stage II colon cancer. (**C**): LAG-3 expression on TILs in the tumor center and its impact on DFS in stage II colon cancer.

The presence of LAG-3 expression on TILs either in the tumor front or tumor center was associated with better DFS (5-year DFS 89.9% (LAG-3 positive TILs) versus 74.7% (LAG-3 negative TILs), HR 0.34, 95% CI 0.14–0.80, *p* = 0.009; Figure 2A). Further analysis demonstrated that the favorable association of LAG-3 positive TILs with DFS was restricted to those that were localized at the tumor front (5-year DFS 91.2% versus 75.2%, HR 0.33, 95% CI 0.13–0.82, *p* = 0.012; Figure 2B). Although statistically not significant, there was a trend towards a longer DFS among LAG-3 positive versus LAG-3 negative TILs in the tumor center (5-year DFS 91.3% versus 81.1%, HR 0.42, 95% CI 0.41–1.24, *p* = 0.106; Figure 2C).

**Figure 3:**
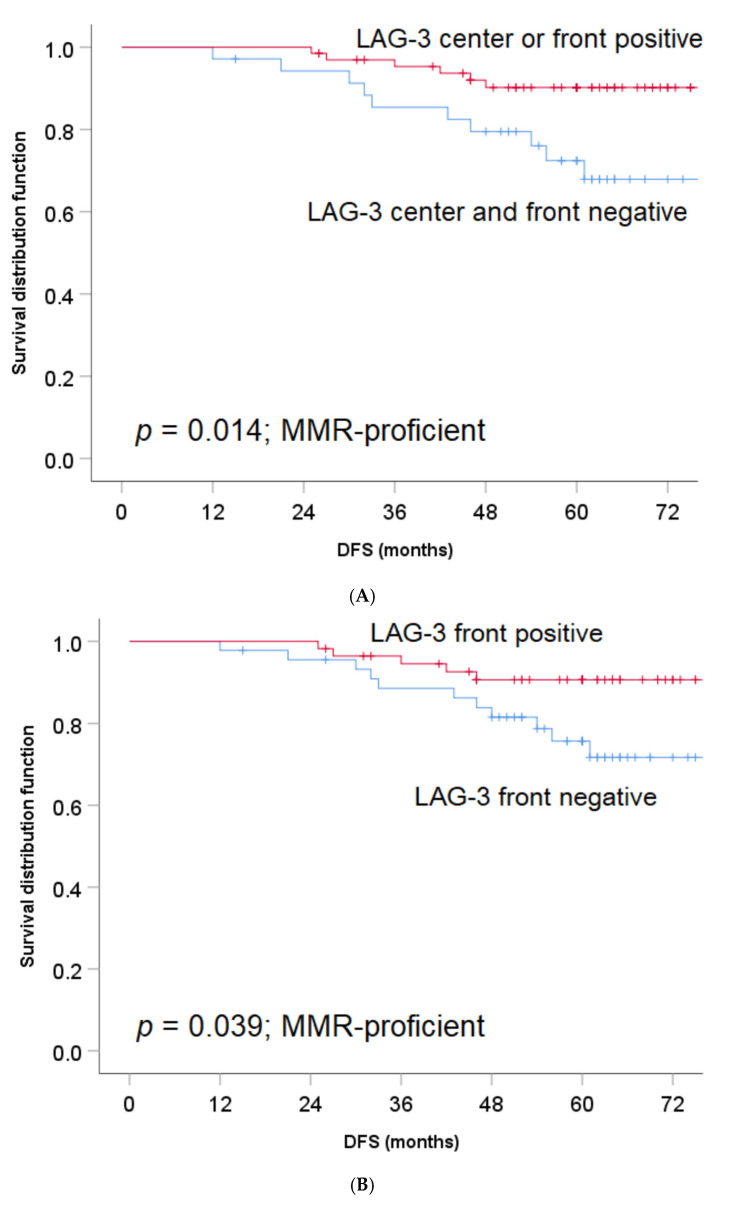

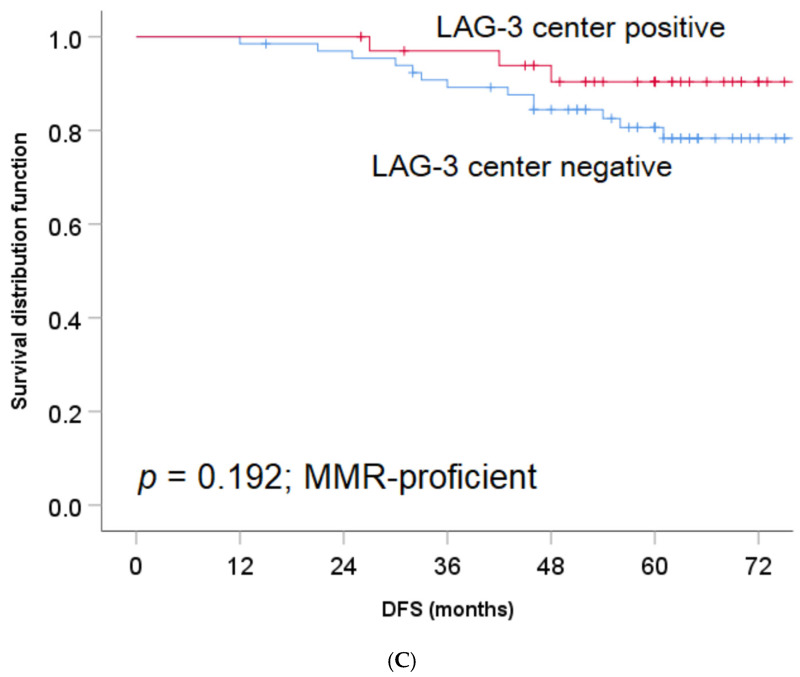
(**A**): The impact of LAG-3 expression on TILs on DFS in patients with stage II MMR-proficient colon cancer (tumor center and tumor front). (**B**): LAG-3 expression on TILs at tumor front and its effect on DFS in stage II MMR-proficient colon cancer. (**C**): LAG-3 expression on TILs in the tumor center and its impact on DFS in stage II MMR-proficient colon cancer.

The favorable association of LAG-3 expression on TILs either in the tumor front or tumor center with the outcome remained significant, even when we considered only MMR-proficient colon cancers (5-year DFS 90.2% versus 67.9%, HR 0.30, 95% CI 0.11–0.83, *p* = 0.014; Figure 3A). Again, the favorable correlation between LAG-3 positive TILs and outcome among MMR-proficient tumors was limited to those localized at the tumor front (Figure 3B), whereas no association with outcome could be observed among LAG-3 positive versus LAG-3 negative TILs in the tumor center (5-year DFS 90.6% versus 75.7%, HR 0.35, 95% CI 0.12–0.99, *p* = 0.039 and 90.4% versus 80.6%, HR 0.44, 95% CI 0.13–1.56, *p* = 0.192, respectively; Figure 3C). 

Due to the low number of events in our cohort of stage II colon cancer, we were not able to conduct a multivariate analysis. However, after adjustment for the pT stage in a bivariate analysis, the favorable effect of LAG-3 expression on DFS remained significant (HR 0.35, 95% CI 0.15–0.83, *p* = 0.017).

## 4. Discussion

To the best of our knowledge, this is the first study evaluating the impact of LAG-3 expression on disease-free survival in stage II colon cancer patients. We demonstrated that LAG-3 expression on TILs was associated with a favorable DFS, especially when LAG-3 positive TILs were identified at the tumor front. 

Our results are consistent with previous findings from other studies. Lee et al. found that patients with stage I–III MMR-deficient colon cancer exhibiting LAG-3 positive TILs had a longer DFS compared to those whose MSI tumors did not contain LAG-3 positive TILs [21]. However, in contrast to our study, Lee et al. included only patients with MSI-high tumors ranging from stage I to stage III, whereas our cohort comprised a homogenous series of patients with stage II colon cancer. Additionally, we did not restrict our analysis to patients with MSI stage II colon cancers alone, but also included patients with MSS tumors, representing the majority of stage II cancer patients.

This is particularly important, as we know from previous studies that not only MSI but also MSS tumors may be enriched by immune infiltrates, representing an immunogenic tumor microenvironment (TME) [22]. In a recently published landmark study comprising tumor tissue samples from 2681 patients with stage I–III colon cancer, Pagès et al. could demonstrate that the numbers of CD3+ and cytotoxic CD8+ T cells in the tumor directly correlated with time to recurrence in both the training and validation cohorts, independent of MSI, T and N stages and other clinicopathological factors. A high Immunoscore was not only associated with a longer time to recurrence (TTR) but also translated into better disease-free survival (DFS) and overall survival (OS). Remarkably, patients with a MSI tumor and a high Immunoscore had a similar outcome compared with those who presented with a MSS tumor and a high Immunoscore. Conversely, patients with MSI tumors and a low Immunoscore exhibited a shorter DFS than those with MSS tumors and a high Immunoscore. In the subgroup of stage II colon cancer patients, these associations remained significant [9].

Thus, further characterization of the TME in both MSI and MSS colon cancers is of utmost importance to gain insight into the complex interplay between immune stimulatory and inhibitory effects within the TME to improve our treatment strategies and to better identify patients who benefit most from systemic treatment.

Similarly, Zhang et al. could demonstrate that LAG-3 expression in a mixed cohort of patients with esophageal squamous cell carcinoma encompassing all stages (I–IV) was associated with improved survival, whereas the favorable effect of high versus low LAG-3 expression on outcome was restricted to stage I–II cancers [23]. In an unselected cohort of patients with stage I –IIIB non-small cell lung cancer, including mainly squamous cell carcinoma and adenocarcinoma but also other histological types such as adenosquamous and large cell carcinoma, LAG-3 expression on TILs was correlated with improved survival [24]. 

Likewise, another study demonstrated that the presence of LAG-3 positive intraepithelial TILs was associated with a longer disease-specific survival among estrogen receptor-negative breast cancer patients [25].

At first glance, it may not seem obvious that the expression of an inhibitory immune-checkpoint molecule such as LAG-3 correlates with a favorable outcome in various solid tumors. Rather, one may assume that the presence of LAG-3 results in increased inhibition of both T cell activation and proliferation and thus contributes to an immune-suppressive tumor microenvironment facilitating tumor growth and metastasis. However, the increased expression of LAG-3 on TILs may not be seen as an independent and ‘isolated’ immune-inhibiting effect but rather be interpreted as an indicator of an enhanced inflammatory immune response, where TILs are stimulated to exert their antitumor response.

Contrary to our results, Chen et al. reported that patients with stage I–IV colorectal cancer exhibiting a high percentage of LAG-3+ cells in the tumor tissue had a shorter survival compared with those with a low percentage of LAG-3+ cells [26]. However, there are several reasons for these opposite findings. First, the study cohort of Chen et al. comprised 108 patients with a mixture of stage I to stage IV colon and rectal cancers, whereas our cohort consisted of stage II patients with colon cancer alone. Interestingly, Chen et al. found that the percentage of LAG-3+ cells in tumor tissues was significantly higher in stage III and IV colorectal cancers compared to that observed in stage I and II cancers. Given that two-thirds of the patients included in the cohort of Chen et al. had stage III and IV colorectal cancers, it might not be surprising that high versus low LAG-3+ expression in the mixed stage I–IV cohort was associated with shorter survival. Additionally, they could demonstrate that a higher percentage of LAG-3+ cells was associated with poor differentiation, lymph node metastasis and invasion [26], whereas no correlation of LAG-3 expression with any of the clinicopathological characteristics such as tumor grade, vascular invasion or tumor budding could be observed in our study cohort of stage II colon cancer. However, we could demonstrate an association between LAG-3 counts and MMR status. Whereas high LAG-3 expression at the tumor front correlated with microsatellite instability (MSI), this association could not be observed in the tumor center. Second, there is no established scoring method for LAG-3. While Chen et al. divided the cohort into tumors with high and low LAG-3-expressing cells [26], we classified our study cohort according to Sobottka et al. [19], into those tumors exhibiting either LAG+ or LAG- TILs at the tumor front or center. Third, there is a significant diversity of antibodies used for immunohistochemistry across several studies. While we used a rabbit monoclonal antibody (Cell Signaling, clone D2G4O Ref 15372), Chen et al. utilized a different LAG-3 antibody from Abcam (Cambridge, MA, USA) without providing any further information [26].

All these different points mentioned above may partly explain the contradictory findings among our studies. Therefore, it is crucial to develop a standard protocol regarding LAG-3 scoring enabling us to better interpret findings from various studies. Saleh et al. could demonstrate that LAG-3 mRNA expression levels in tumor tissues versus paired normal tissues of colorectal cancer patients were approximately similar [27]. Additionally, Toor et al. could demonstrate in a small and mixed cohort of stage I–IV colorectal cancer patients using a flow cytometry assay that LAG-3 expression on peripheral mononuclear leukocytes was significantly lower compared to the levels observed on both tumor-infiltrating lymphocytes (TILs) and lymphocytes from adjacent normal colon tissue (NILs). Again, no significant difference in LAG-3 expression could be detected between TILs and NILs [28].

With the introduction of immune checkpoint therapies such as PD1 and PD-L1 inhibitors, the prognosis of cancer patients with various malignancies such as melanoma [29], non-small cell lung cancer [30], renal carcinoma [31], urothelial carcinoma [32], head and neck carcinoma [33] and Hodgkin lymphoma has significantly improved [34]. Currently, there are several clinical trials evaluating the effect of LAG-3 inhibitors in different tumor types [35,36]. Therefore, both the diagnostic and therapeutic value of LAG-3 might be evolving in the near future. Whereas immune checkpoint therapy is associated with improved tumor control and longer survival in patients with MSI metastatic colorectal cancer (mCRC), and thus represents the standard of care treatment [37,38], its effect on outcome in patients with microsatellite stable (MSS) mCRC, who represent 95% of all patients with mCRC, is so far disappointing [39]. Additionally, the role of immune checkpoint therapy as part of the adjuvant treatment strategy in both MSS and MSI stage II and stage III still remains elusive, with several studies ongoing [40,41].

The limitations of this study are its monocentric retrospective design, the small sample size and the lack of an independent clinical validation cohort. Moreover, we restricted our analysis to the immunohistochemical assessment of LAG-3 without using further assays such as in situ hybridization or assessment of ELISA serum LAG-3 concentrations. The latter could not be done due to the lack of blood samples. Additionally, the lack of a consensus guideline regarding immunohistochemical LAG-3 scoring makes it challenging to draw any cross-comparisons between studies. However, our strengths are that we restricted our analysis to a well-defined homogenous cohort of patients with stage II colon cancer. In accordance with Sobottka et al., we performed a binary scoring algorithm for LAG-3 expression. By using a dichotomous scoring method rather than a quantitative scoring method with different thresholds, we may minimize the interobserver variability, increase the reproducibility and facilitate further clinical validation studies in other patient cohorts. Given the lack of a general scoring guideline, this simple binary assessment of LAG-3 allows for efficient, cost-effective and easily reproducible scoring that might be implemented in the diagnostic algorithm of stage II colon cancer, enabling clinicians to decide whether a patient should undergo adjuvant chemotherapy.

## 5. Conclusions

In conclusion, we were able to demonstrate that LAG-3 expression on TILs at the tumor front of stage II colon cancers was associated with better outcomes in both the overall stage II cohort and within the subgroup of stage II MSS tumors. Therefore, LAG-3 might serve as a potential prognostic biomarker. However, further studies are needed to explore whether the assessment of LAG-3 in stage II colon cancer may help us to identify those patients who derive the most benefit from adjuvant chemotherapy.

## Figures and Tables

**Table 1:** Association of LAG-3 (combined tumor front and center) with clinicopathological features on the stage II colon cancer cohort.

Feature		Combined		*p*-Value
		Front and Center negative	Front or Center positive	
Age, years (*n* = 141)	Mean ± SD	69.5 ± 13.8	67.5 ± 15.7	0.493
Gender (*n* = 142)	Male	24 (52.2)	58 (60.4)	0.352
	Female	22 (47.8)	38 (39.6)	
pT (*n* = 141)	pT3	40 (88.9)	81 (84.4)	0.474
	pT4	5 (11.1)	15 (15.6)	
Tumor grade (*n* = 134)	G1/G2	43 (97.8)	79 (87.8)	0.058
	G3	1 (2.2)	11 (12.2)	
EMVI (*n* = 135)	V0	37 (88.1)	81 (87.1)	0.871
	V1	5 (11.9)	12 (12.9)	
Tumor location (*n* = 139)	Left	26 (59.1)	47 (49.5)	0.291
	Right	18 (40.9)	48 (50.5)	
Tumor budding (ITBCC) (*n* = 142)	Mean ± SD	11.1 ± 11.4	11.9 ± 10.8	0.408
MMR status (*n* = 134)	Proficient	35 (87.5)	66 (70.2)	**0.034**
	Deficient	5 (12.5)	28 (29.8)	

Data are presented as *n* (%), unless otherwise stated. Abbreviations: SD = standard deviation; pT = pathological T stage (TNM classification system); EMVI = extramural vascular invasion; ITBCC = International Tumor Budding Consensus Conference. Bold indicates statistical significant *p*-values

**Table 2:** Association of LAG-3 (front/tumor center) with clinicopathological features on the stage II colon cancer cohort.

Feature		Front			Center		
		Negative	Positive	*p*-value	Negative	Positive	*p*-value
Age, years (*n* = 141)	Mean ± SD	69.8 ± 13.9	67.1 ± 14.6	0.316	68.4 ± 14.0	67.7 ± 14.9	0.784
Gender (*n* = 142)	Male	31 (54.4)	51 (60.0)	0.507	52 (56.5)	28 (58.3)	0.837
	Female	26 (45.6)	34 (40.0)		40 (43.5)	20 (41.7)	
pT (*n* = 141)	pT3	48 (85.7)	73 (85.9)	0.978	78 (85.7)	41 (85.4)	0.962
	pT4	8 (14.3)	12 (14.1)		13 (4.3)	7 (14.6)	
Tumor grade (*n* = 134)	G1/G2	52 (96.3)	70 (87.5)	0.08	86 (95.6)	36 (83.7)	**0.021**
	G3	2 (3.7)	10 (12.5)		4 (4.4)	7 (16.3)	
EMVI (*n* = 135)	V0	46 (88.5)	72 (86.8)	1.0	75 (86.2)	42 (91.3)	0.39
	V1	6 (11.5)	11 (13.3)		12 (13.8)	4 (8.7)	
Tumor location (*n* = 139)	Left	33 (60.0)	40 (47.6)	0.168	51 (57.3)	21 (43.8)	0.123
	Right	22 (40.0)	44 (52.4)		38 (42.7)	27 (56.3)	
Tumor budding (ITBCC) (*n* = 142)	Mean ± SD	11.2 ± 11.6	11.9 ± 10.5	0.227	12.5 ± 11.7	10.0 ± 9.3	0.142
MMR status (*n* = 134)	Proficient	45 (86.5)	56 (68.3)	**0.017**	66 (75.0)	35 (76.1)	0.89
	Deficient	7 (13.5)	26 (31.7)		22 (25.0)	11 (23.9)	

Data are presented as *n* (%), unless otherwise stated. SD = standard deviation; pT = pathological T stage (TNM classification system); EMVI = extramural vascular invasion; ITBCC = International Tumor Budding Consensus Conference. Bold indicates statistical significant *p*-values.

## Data Availability

The data presented in this study are available from the corresponding author upon reasonable request.

## Supplementary Materials

The following are available online at https://www.mdpi.com/article/10.3390/jpm11080749/s1, Table S1: Baseline characteristics.
