# Supplementary material for: LAG-3 Expression Predicts Outcome in Stage II Colon Cancer
Source: J Pers Med. 2021 Jul 30;11(8):749. doi: 10.3390/jpm11080749 (PMC8398428; doi:10.3390/jpm11080749)
Supplement: Supplementary file 1 [file jpm-11-00749-s001.zip › jpm-1322158-supplementary.pdf]

Supplementary Table S1. Baseline characteristics

**Feature**		**Total**
Age, years (n=141)	Median	70 (range, 24-98)
Gender (n=142)	Male	82 (57.8)
	Female	60 (42.2)
pT (n=141)	pT3	121 (85.8)
	pT4	20 (14.2)
Tumor grade (n=134)	G1/G2	122 (91.0)
	G3	12 (9.0)
EMVI (n=135)	V0	118 (87.4)
	V1	17 (12.6)
Tumor location (n=139)	Left	73 (52.5)
	Right	66 (47.5)
Lymph node yield (n=142)	≥12	124 (87.3)
	<12	18 (12.7)
Tumor budding (ITBCC) (n=142)	Median	10 (range, 0-74)
MMR status (n=134)	deficient	33 (24.6)
	proficient	101 (75.4)

Data are presented as n (%), unless otherwise stated. Abbreviations: pT = pathological T stage (TNM classification system); EMVI = extramural vascular invasion; ITBCC = International Tumor Budding Consensus Conference.
